# Prevalence of *Neisseria gonorrhoeae*, *Ureaplasma urealyticum*, and *Chlamydia trachomatis* infections in 37,680 patients in Guangzhou, China

**DOI:** 10.1128/spectrum.00156-26

**Published:** 2026-10-15

**Authors:** Jiaying Li, Meiling Liu, Guoyi Weng, Wuwei Zeng, Bangming Xu, Donglin Cao

**Affiliations:** 1 The Affiliated Guangdong Second Provincial General Hospital of Jinan University 485285, Guangzhou, Guangdong, China; 2 Department of Blood Transfusion, The Sixth Affiliated Hospital, Sun Yat-sen University 373651, Guangzhou, Guangdong, China; 3 Biomedical Innovation Center, The Sixth Affiliated Hospital, Sun Yat-sen University 373651, Guangzhou, Guangdong, China; MultiCare Health System, Tacoma, Washington, USA

**Keywords:** *Neisseria gonorrhoeae*, *Ureaplasma urealyticum*, *Chlamydia trachomatis*, sexually transmitted infections

## Abstract

To refine the epidemiological profile of sexually transmitted infections (STIs) in Guangzhou, this study analyzed *Neisseria gonorrhoeae* (NG), *Ureaplasma urealyticum* (UU), and *Chlamydia trachomatis* (CT) infections among 37,680 patients treated at the Affiliated Guangdong Second Provincial General Hospital of Jinan University from 2020 to 2024. Overall prevalence was 15.48% for NG, 30.91% for UU, and 4.78% for CT. NG and CT prevalences were both significantly higher in males than in females (NG: 25.07% vs 2.86%; CT: 5.33% vs 4.28%), whereas UU prevalence was higher in females (41.67% vs 24.96%). Age-specific patterns revealed distinct trends. NG prevalence in males peaked in the ≤20 age group (48.34%) and declined with age. UU prevalence was highest in females ≤20 years (60.00%). CT prevalence peaked in youth ≤20 years (25% and 30.34% for males and females, respectively), with lower rates observed in subsequent age groups. Regarding co-infections, UU+CT was the most frequent (5.00%). NG+UU and NG+CT co-infections were male-predominant, whereas UU+CT was significantly higher in females ≤20 years (36.00% vs 13.95%). *O*/*E* and OR analyses suggested a synergistic interaction for UU+CT in both sexes aged ≤30 years. Triple infection (NG+UU+CT) remained below 1% and showed no significant sex difference. Stratified by clinical presentation, NG and CT infections in males were highly concentrated in the urogenital inflammation group, demonstrating a symptom-driven pattern. In females, CT infection was prevalent in the urogenital inflammation (7.37%), others (7.11%), and screening and asymptomatic examination (5.86%) groups, and NG was detected in both the inflammation (3.46%) and screening (3.61%) groups, indicating a coexistence of symptomatic and occult infections. UU showed consistently high prevalence across all female clinical groups (32.50%–46.07%). Our study provided evidence for tailored STI control strategies.

**IMPORTANCE:**

This study systematically characterized the epidemiological profile of *Neisseria gonorrhoeae* (NG), *Ureaplasma urealyticum* (UU), and *Chlamydia trachomatis* (CT) infections in Guangzhou, revealing distinct sex- and age-specific patterns, identifying UU+CT as the predominant co-infection with a synergistic interaction in individuals aged ≤30 years, and demonstrating a symptom-driven pattern for NG and CT in males versus a dual burden of symptomatic and occult infections in females. These findings fill a gap in the epidemiological data in South China and support gender-specific screening strategies that distinguish symptomatic management in males from proactive case-finding in females.

## INTRODUCTION

Sexually transmitted infections (STIs) pose a significant global public health challenge. *Neisseria gonorrhoeae* (NG), *Ureaplasma urealyticum* (UU), and *Chlamydia trachomatis* (CT) are common sexually transmitted pathogens. These infections can lead to severe consequences for an individual’s reproductive health, including urethritis, vaginitis, cervicitis, endometritis, and even infertility (1, 2). NG is a Gram-negative diplococcus and the primary causative agent of gonorrhea, which is predominantly transmitted through sexual contact. Gonorrhea ranks among the most common sexually transmitted infections worldwide, with particularly high incidence rates among young adults (3). Infections with NG can lead to urethritis, cervicitis, and pelvic inflammatory disease (4). In severe cases, complications may include infertility and ectopic pregnancy (2). The increasing antibiotic resistance observed in NG has posed significant challenges for clinical management (5). In recent years, UU has been one of the main pathogens causing chronic non-gonococcal urethritis (6). UU infections occur predominantly among young adults, often following unprotected sexual contact. Beyond non-gonococcal urethritis, UU infection can lead to a range of reproductive health issues, including pelvic inflammatory disease, infertility in women of childbearing age, spontaneous abortion, and fetal growth restriction (7, 8). CT infection can cause urethritis, cervicitis, and pelvic inflammatory disease, with severe cases leading to infertility and ectopic pregnancy (2). Often asymptomatic or mild, CT infections are frequently overlooked, resulting in chronic infection and complications. Emerging evidence suggests a potential association between chronic CT infection and an elevated risk of cervical carcinogenesis, which may be mediated through the induction of persistent local inflammation, promotion of cellular damage, and potential upregulation of human papillomavirus oncogenes, thereby creating a microenvironment conducive to tumorigenesis (9–12).

The prevalence of NG, UU, and CT infections varies significantly across regions, genders, and age groups. The positivity rate for NG is typically higher in males than in females, whereas a significantly higher UU positivity rate is observed in females. Age is also a significant factor influencing prevalence rates, with younger individuals, particularly those under 21 years of age, typically exhibiting higher infection rates. Furthermore, co-infections among NG, UU, and CT have been demonstrated to be closely associated with increased disease severity, treatment challenges, and poorer clinical outcomes (13–16).

Understanding the epidemiological characteristics and co-infection patterns of NG, UU, and CT is crucial for developing scientific and effective STI prevention and control strategies. Nevertheless, large-scale epidemiological data from South China remain scarce. To address this gap, this study analyzed the infection patterns of NG, UU, and CT among 37,680 patients treated at the Affiliated Guangdong Second Provincial General Hospital of Jinan University from 2020 to 2024, aiming to characterize the pathogen distribution across genders, age groups, and clinical diagnoses, and to explore co-infection patterns. The findings will provide a scientific basis for developing STI prevention and control strategies and offer valuable references for clinical diagnosis and treatment in South China.

## MATERIALS AND METHODS

### Study population

The analysis included data from the period between January 2020 and December 2024. A total of 37,680 eligible patients were included, with all data originating from the hospital’s centralized Laboratory Information System (LIS). The inclusion criteria were as follows: (i) initial clinical visit; (ii) nucleic acid test result available for at least one pathogen (NG, UU, or CT), which was performed based on clinically indicated testing ordered by attending physicians in accordance with the national diagnostic criteria for sexually transmitted diseases issued by the National Health Commission of China and relevant clinical diagnosis and treatment guidelines from the Chinese Center for Disease Control and Prevention and the Chinese Medical Association. These indications encompassed the presence of genitourinary symptoms or signs, screening of high-risk populations or individuals undergoing assisted reproduction, and opportunistic screening prompted by patient request or risk factors identified during medical history taking; (iii) availability of complete clinical information; and (iv) collection of a qualified specimen. The cohort comprised 17,852 males aged 13–92 (mean age: 33.91 ± 7.13) and 19,828 females aged 10–91 (mean age: 33.46 ± 7.19). Demographic information and principal clinical diagnoses at the time of visit were retrospectively extracted from the LIS. To analyze the associations between pathogen infections and specific health conditions, patients were categorized into one of six mutually exclusive groups based on their principal clinical diagnoses. A predefined hierarchy of diagnoses was sequentially applied to determine the primary group for patients with multiple clinical diagnoses: (i) urogenital inflammation; (ii) history of adverse pregnancy and childbirth; (iii) infertility; (iv) non-inflammatory genitourinary diseases; (v) screening and asymptomatic examination; and (vi) others. The “others” category included patients with endocrine, dermatological, or digestive diseases who were tested via hospital-wide screening or opportunistic testing protocols.

1. Urogenital inflammation
  Males included urethritis, epididymitis, spermatocystitis, prostatitis, or balanitis.
  Females included urethritis, vaginitis, cervicitis, pelvic inflammatory disease, or adnexitis.
2. History of adverse pregnancy and childbirth
  Males included partner’s history of adverse pregnancy outcomes.
  Females included pregnancy loss-related conditions, including spontaneous abortion (threatened, inevitable, missed, and complete), recurrent spontaneous abortion, ectopic pregnancy, induced abortion, intrauterine adhesion post-abortion, and immune-mediated abortion.
3. Infertility
  Males included male infertility, oligozoospermia, asthenozoospermia, teratozoospermia, azoospermia, leukocytospermia, seminal fluid abnormality, cryptorchidism, testicular atrophy/agenesis, or chromosomal abnormality.
  Females included female infertility, ovulation disorder, hydrosalpinx, tubal obstruction, tubal adhesion, intrauterine adhesion, or chromosomal abnormality.
4. Non-inflammatory genitourinary diseases
  Males included benign prostatic hyperplasia, urethral stricture, phimosis/redundant prepuce, or varicocele.
  Females included uterine myoma (leiomyoma), adenomyosis, endometrial polyp, endometrial hyperplasia, endometriosis, uterine prolapse, abnormal uterine bleeding, cervical intraepithelial neoplasia, cervical polyp, menstrual irregularity, dysmenorrhea, Nabothian cyst, or polycystic ovary syndrome.
5. Screening and asymptomatic examination
  General health check-up, fertility assessment, premarital screening, or a routine physical examination without specific urogenital complaints.
6. Others
  Fever of unknown origin, upper respiratory tract infection, pneumonia, cough, abdominal pain of unknown origin, digestive system diseases, dermatitis, urticaria, acne, pruritus, alopecia, diabetes mellitus, thyroid disorders, hyperuricemia, obesity, osteoporosis, hypertension, coronary heart disease, anemia, thalassemia, leukemia, headache, dizziness, sleep disorder, anxiety, arthralgia, or a personal history of malignancy.

### Specimen collection

Patients were instructed to refrain from urinating for at least 2 h before sampling. Male patients: the urethral meatus was cleansed with sterile saline. A sterile male swab was inserted 2–3 cm into the urethra, rotated firmly 1–2 times, withdrawn, and immediately placed into a sterile collection tube, which was securely sealed for transport. Female patients: excessive cervical secretions were first cleared. A sterile female swab was then inserted 2–3 cm into the endocervical canal, rotated firmly 1–2 times, and withdrawn. The collected swab was placed in a sterile collection tube and securely sealed for transport.

### Specimen testing

Pathogen detection was performed using real-time quantitative polymerase chain reaction (qPCR) on Applied Biosystems 7500 (Thermo Fisher Scientific, USA) with commercial, *in vitro* diagnostic-approved kits approved for clinical diagnostics from Sansure Biotech (Hunan, China), including the UU (Cat#20153400087), NG (Cat#20153400086), and CT (Cat#20153400084) Nucleic Acid Detection Kit. All assays were performed strictly according to the manufacturer’s instructions and the laboratory’s Standard Operating Procedures. For sample preparation, 1 mL of sterile physiological saline was added to each collection tube, followed by vigorous vortexing to ensure complete homogenization. The resulting supernatant was transferred to 1.5 mL sterile microcentrifuge tubes for further processing. For PCR setup, 5 μL of sample release agent was added to each reaction tube and mixed by pipetting 3–5 times, followed by 40 μL of PCR master mix. The tubes were briefly centrifuged before PCR amplification.

### Result interpretation

For NG and CT, samples with a cycle threshold (Ct) value ≤38 and a characteristic S-shaped amplification curve were reported as NG or CT DNA-positive. UU positivity was defined as a Ct value ≤38.5 with a clear S-shaped amplification curve. These thresholds were established based on the manufacturers’ recommendations and internal validation.

Test-specific prevalence was calculated for each pathogen and their combinations. For each category, the rate was defined as


$$Prevalence=\frac{Number\;of\;cases\;positive\;for\;all\;pathogens\;in\;the\;category}{Total\;number\;of\;cases\;tested\;for\;all\;pathogens\;in\;the\;category\;}\times 100\%.$$

For each infection category, the denominator comprised individuals tested for all pathogens in that category, and the numerator comprised those positive for all of them.

### Statistical analysis

Statistical analyses were performed using IBM SPSS Statistics (Version 27.0, USA). Categorical data were presented as counts (*n*) and corresponding percentages (%). Binomial exact methods were employed to calculate 95% confidence intervals (CIs) for infection prevalence. Between-group differences were evaluated using chi-square tests. Pearson chi-square was applied when all expected cell frequencies were ≥5; the likelihood-ratio chi-square was used when expected cell frequencies fell below 5. For post hoc pairwise comparisons, Bonferroni correction was applied to adjust for multiple testing. All tests were two-sided, and *P* < 0.05 was considered statistically significant. Interactions between two pathogens were assessed on the multiplicative scale. Single infection rates were calculated for each pathogen. Based on the product rule of independent probabilities, the expected co-infection rate for each pathogen pair was computed as

*P*_expected_ (Pathogen A^+^ ∩ Pathogen B^+^) = *P* (Pathogen A^+^) × *P* (Pathogen B^+^),

where the superscripts “+” and “−” denote positive and negative test results, respectively.

The observed-to-expected coinfection ratio (*O*/*E* ratio) was calculated as


$$O/E=\frac{Observed\;coinfection\;cases}{Expected\;coinfection\;cases}.$$

An *O*/*E* ratio >1 indicates co-occurrence exceeding expectation under independence, whereas an *O*/*E* ratio <1 indicates co-occurrence below expectation. To formally test for interaction on the multiplicative scale, stratum-specific odds ratios (ORs) with 95% CIs were estimated. For each pathogen pair, a 2 × 2 contingency table (Pathogen A^±^ × Pathogen B^±^) was constructed, and the OR was computed as


$$OR=\frac{\left(a\times d\right)}{\left(b\times c\right)},$$

where *a*, *b*, *c*, and *d* represent the cell counts of the 2 × 2 table (Pathogen A^±^ × Pathogen B^±^).

A synergistic interaction was defined as OR >1 with 95% CI excluding 1; an antagonistic interaction as OR <1 with 95% CI excluding 1; and no significant interaction if the 95% CI included 1. These are statistical interactions and do not presuppose a biological mechanism.

## RESULTS

### Overall prevalence of NG, UU, and CT in the study population

A total of 37,680 cases (age range: 10–92 years, average age: 33.67 ± 7.17 years) were enrolled in the study, including 17,852 males and 19,828 females. The overall prevalence of NG, UU, and CT was 15.48% (766/4,947), 30.91% (1632/5,280), and 4.78% (1765/36,939), respectively. The most frequent co-infection was UU+CT (5.00% [236/4,717]), whereas NG+UU (3.06% [72/2,353]), NG+CT (2.38% [113/4,745]), and NG+UU+CT (0.77% [18/2,329]) were less common (Table 1).

**TABLE 1:** Overall prevalence of NG, UU, and CT in the study population

Category of detection	Positive cases/*n*	Prevalence (%)	95% CI
NG	766/4,947	15.48	14.48–16.49
UU	1,632/5,280	30.91	29.66–32.16
CT	1,765/36,939	4.78	4.56–5.00
NG+UU	72/2,353	3.06	2.36–3.76
NG+CT	113/4,745	2.38	1.95–2.82
UU+CT	236/4,717	5.00	4.38–5.63
NG+UU+CT	18/2,329	0.77	0.42–1.13

### Sex- and age-specific prevalence of pathogens

Sex-specific analysis revealed distinct patterns of pathogen prevalence and co-infection between males and females. The prevalence of NG was significantly higher in males (25.07% [705/2,812]) than in females (2.86% [61/2,135]). In contrast, the prevalence of UU was significantly higher in females (41.67% [783/1,879]) compared to males (24.96% [849/3,401]). A significantly higher prevalence of CT was observed in males (5.33% [930/17,443]) compared to females (4.28% [835/19,496]).

Regarding co-infections, the prevalence of NG+UU (3.86% [60/1,554] vs 1.50% [12/799]) and NG+CT (3.42% [93/2,722] vs 0.99% [20/2,023]) was significantly higher in males than in females. Conversely, the prevalence of UU+CT co-infection was slightly higher in females (5.42% [89/1,641]) than in males (4.78% [147/3,076]), but this difference was not statistically significant. Similarly, the prevalence of triple co-infection (NG+UU+CT) had no significant sex-based difference (0.90% [14/1,548] in males vs 0.51% [4/781] in females) (Table 2).

**TABLE 2:** Sex-specific prevalence and co-infection patterns of NG, UU, and CT

Category of detection	Males			Females			χ2	*P* value
	Positive cases/*n*	Prevalence (%)	95% CI	Positive cases/*n*	Prevalence (%)	95% CI		
NG	705/2,812	25.07	23.47–26.67	61/2,135	2.86	2.15–3.56	457.614	<0.001
UU	849/3,401	24.96	23.51–26.42	783/1,879	41.67	39.44–43.90	158.210	<0.001
CT	930/17,443	5.33	5.00–5.67	835/19,496	4.28	4.00–4.57	22.254	<0.001
NG+UU	60/1,554	3.86	2.90–4.82	12/799	1.50	0.66–2.35	9.901	0.002
NG+CT	93/2,722	3.42	2.73–4.10	20/2,023	0.99	0.56–1.42	29.428	<0.001
UU+CT	147/3,076	4.78	4.02–5.53	89/1,641	5.42	4.33–6.52	0.935	0.333
NG+UU+CT	14/1,548	0.90	0.43–1.38	4/781	0.51	0.01–1.01	1.041	0.308

**Fig 1:**
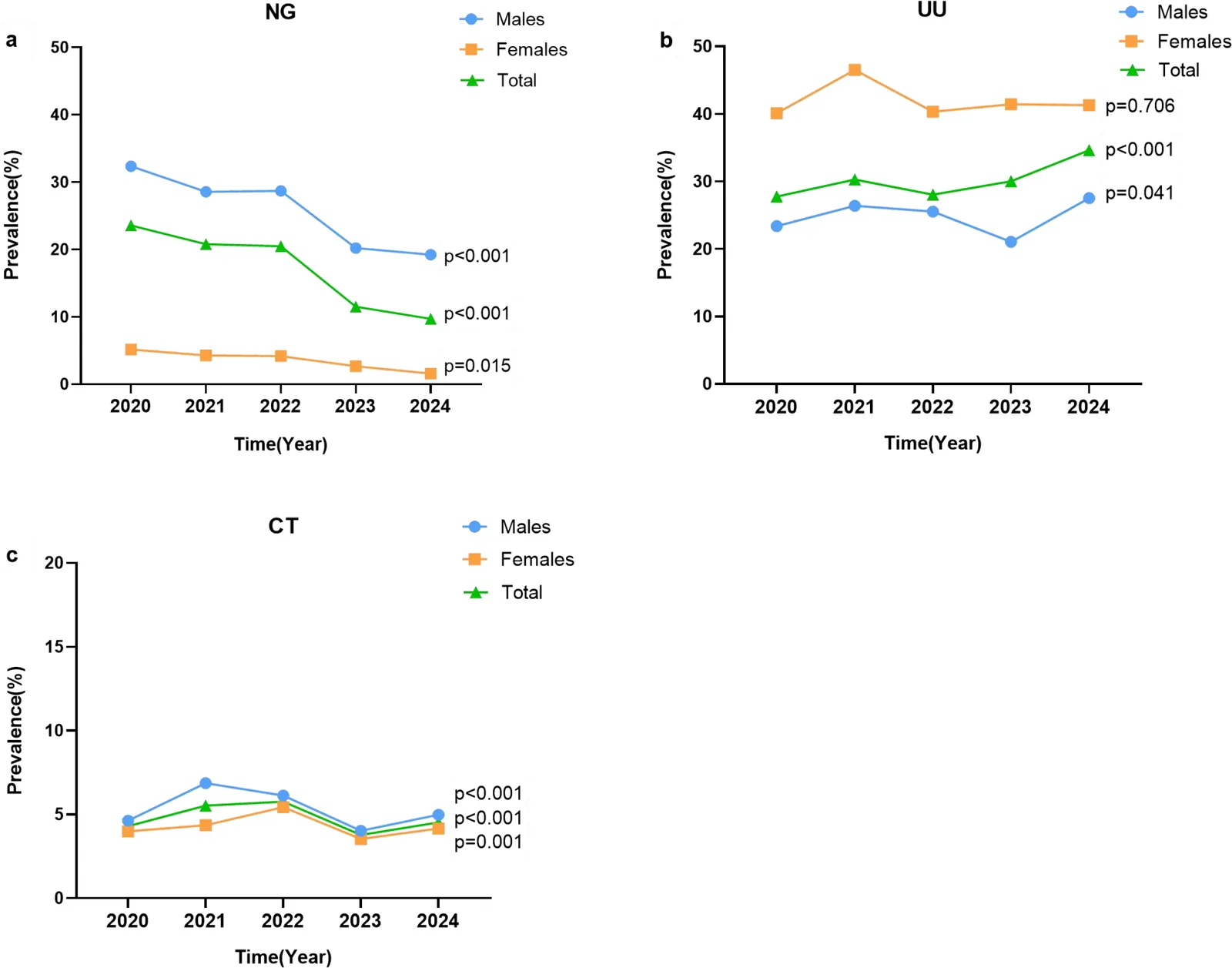
Temporal trends in prevalence of NG (**a**), UU (**b**), and CT (**c**), 2020–2024.

From 2020 to 2024, NG prevalence showed an overall decline in both males and females. Male prevalence decreased from 32.35% (132/408) to 19.21% (136/708), and female prevalence decreased from 5.13% (10/195) to 1.57% (13/830). Prevalence differed significantly across study years in both sexes. Moreover, the male-dominated epidemiological pattern remained unchanged throughout the study period (Fig. 1a). The prevalence of UU in females remained relatively stable, ranging from 40.10% (79/197) to 46.52% (87/187), with minor fluctuations, and no significant difference was found across the years. In contrast, the prevalence in males fluctuated between 21.04% (141/670) and 27.53% (220/799). A significant difference in prevalence across the study years was observed in males and in the overall population (Fig. 1b). CT prevalence in males ranged from 4.03% (161/3,993) to 6.86% (270/3,936), and in females ranged from 3.54% (144/4,070) to 5.44% (198/3,639). Notably, the rates for both sexes reached their lowest point in 2023. Significant differences in prevalence were observed across the study years for both males and females (Fig. 1c).

Comparison of the prevalence between males and females across different age groups revealed distinct sex-specific patterns for the three pathogens. NG exhibited a significantly higher prevalence in males than in females across all age groups, with the most pronounced disparity observed in the ≤20 age group (48.34% [73/151] vs 9.28% [9/97]). In contrast, UU demonstrated a significantly higher prevalence in females aged less than or equal to 50 years old, peaking at 60.00% (36/60) in females aged ≤20 years. Moreover, CT showed significantly higher male prevalence in the 21–30 and 31–40 age groups (Table 3).

**TABLE 3:** Sex-specific prevalence of NG, UU, and CT by age group

Category of detection	Age (years)	Males			Females			χ2	*P* value
		Positive cases/*n*	Prevalence (%)	95% CI	Positive cases/*n*	Prevalence (%)	95% CI		
NG	≤20	73/151	48.34	40.28–56.41	9/97	9.28	3.40–15.16	40.726	<0.001
	21–30	355/1,255	28.29	25.79–30.78	37/886	4.18	2.86–5.50	201.856	<0.001
	31–40	201/911	22.06	19.37–24.76	8/733	1.09	0.34–1.85	160.997	<0.001
	41–50	60/337	17.80	13.70–21.91	4/308	1.30	0.03–2.57	49.050	<0.001
	≥51	16/158	10.13	5.37–14.88	3/111	2.70	0.00–5.77	5.474	0.019
UU	≤20	46/133	34.59	26.40–42.78	36/60	60.00	47.24–72.76	10.928	<0.001
	21–30	371/1,498	24.77	22.58–26.95	300/692	43.35	39.65–47.05	76.942	<0.001
	31–40	286/1,193	23.97	21.55–26.40	282/706	39.94	36.32–43.56	53.959	<0.001
	41–50	101/391	25.83	21.47–30.19	119/286	41.61	35.86–47.36	18.744	<0.001
	≥51	45/186	24.19	17.98–30.41	46/135	34.07	25.98–42.17	3.760	0.053
CT	≤20	47/188	25.00	18.75–31.25	71/234	30.34	24.41–36.28	1.477	0.224
	21–30	446/5,644	7.90	7.20–8.61	424/6,848	6.19	5.62–6.76	13.973	<0.001
	31–40	333/8,858	3.76	3.36–4.16	249/9,571	2.60	2.28–2.92	20.161	<0.001
	41–50	81/2,334	3.47	2.73–4.21	76/2,393	3.18	2.47–3.88	0.319	0.572
	≥51	23/419	5.49	3.30–7.68	15/450	3.33	1.67–5.00	2.412	0.120

Comparative analysis of co-infection patterns revealed that the sex-specific prevalence differences were not universally maintained in mixed infections. The prevalence of NG+UU co-infection showed no significant differences between sexes across all age groups. NG+CT co-infection was significantly more prevalent in males aged ≤40 years. In contrast, UU+CT co-infection demonstrated a significant female predominance exclusively in the ≤20 age group (36.00% [18/50] in females vs 13.95% [18/129] in males), with no significant sex-based differences in other age groups. The overall prevalence of triple co-infection (NG+UU+CT) was low (<1%) and did not differ significantly by sex (Table 4).

**TABLE 4:** Sex-specific prevalence of co-infections by age group^*a*^

Category of detection	Age (years)	Males			Females			χ2	*P* value
		Positive cases/*n*	Prevalence (%)	95% CI	Positive cases/*n*	Prevalence (%)	95% CI		
NG+UU	≤20	19/101	18.81	11.06–26.57	2/27	7.41	0.00–17.96	2.342	0.126
	21–30	25/737	3.39	2.08–4.70	7/321	2.18	0.57–3.79	1.119	0.290
	31–40	9/480	1.88	0.66–3.09	2/284	0.70	0.00–1.68	1.918	0.166
	41–50	6/151	3.97	0.82–7.12	1/122	0.82	0.00–2.44	3.044	0.081
	≥51	1/85	1.18	0.00–3.52	0/45	0.00	0.00–0.00	0.854	0.355
NG+CT	≤20	17/142	11.97	6.57–17.38	3/84	3.57	0.00–7.62	4.617	0.032
	21–30	46/1,213	3.79	2.72–4.87	10/827	1.21	0.46–1.96	12.290	<0.001
	31–40	19/882	2.15	1.19–3.11	3/714	0.42	0.00–0.90	8.727	0.003
	41–50	9/330	2.73	0.96–4.49	3/297	1.01	0.00–2.15	2.455	0.117
	≥51	2/155	1.29	0.00–3.09	1/101	0.99	0.00–2.95	0.049	0.826
UU+CT	≤20	18/129	13.95	7.89–20.01	18/50	36.00	22.22–49.78	10.901	<0.001
	21–30	82/1,354	6.06	4.78–7.33	44/592	7.43	5.31–9.55	1.288	0.256
	31–40	39/1,077	3.62	2.50–4.74	17/636	2.67	1.42–3.93	1.137	0.286
	41–50	4/353	1.13	0.02–2.24	7/251	2.79	0.74–4.84	2.211	0.137
	≥51	4/163	2.45	0.05–4.85	3/112	2.68	0.00–5.72	0.013	0.908
NG+UU+CT	≤20	8/101	7.92	2.56–13.28	0/26	0.00	0.00–0.00	3.801	0.051
	21–30	5/734	0.68	0.08–1.28	2/312	0.64	0.00–1.53	0.005	0.942
	31–40	0/478	0.00	0.00–0.00	1/280	0.36	0.00–1.06	1.994	0.158
	41–50	1/150	0.67	0.00–1.98	1/120	0.83	0.00–2.48	0.025	0.874
	≥51	0/85	0.00	–	0/43	0.00	–	–	–

^*a*^ "–" indicates that the 95% CI, χ² value and *P* value were not estimable due to the presence of groups containing zero observed cases.

Analysis of the age-specific trends in pathogen prevalence revealed distinct patterns. The prevalence of NG exhibited a significant declining trend with age in males, decreasing from 48.34% (73/151) in the ≤20 age group to 10.13% (16/158) in the ≥51 age group. In females, however, the NG prevalence was 9.28% (9/97) in the ≤20 age group, declined to its lowest point of 1.09% (8/733) in the 31–40 age group, and increased to 2.70% (3/111) in the ≥51 age group. The prevalence of UU remained relatively stable across age groups, with that in females consistently higher than in males. The age-specific trend for UU was significant in females but was not significant in males. The age-specific prevalence of CT showed significant variation in both males and females. CT prevalence peaked in males aged ≤20 years at 25.00% (47/188) and dropped to 3.76% (333/8,858) in those aged 31–40 years and 3.47% (81/2,334) in those aged 41–50 years. In females, CT prevalence also decreased markedly from 30.34% (71/234) in the ≤20 age group to 2.60% (249/9,571) in the 31–40 age group and 3.18% (76/2,393) in the 41–50 age group (Table 5).

**TABLE 5:** Age-specific prevalence of NG, UU, and CT, stratified by sex

Sex	Age (years)	NG			UU			CT		
		Positive cases/*n*	Prevalence (%)	95% CI	Positive cases/*n*	Prevalence (%)	95% CI	Positive cases/*n*	Prevalence (%)	95% CI
Males	≤20	73/151	48.34	40.28–56.41	46/133	34.59	26.40–42.78	47/188	25.00	18.75–31.25
	21–30	355/1,255	28.29	25.79–30.78	371/1,498	24.77	22.58–26.95	446/5,644	7.90	7.20–8.61
	31–40	201/911	22.06	19.37–24.76	286/1,193	23.97	21.55–26.40	333/8,858	3.76	3.36–4.16
	41–50	60/337	17.80	13.70–21.91	101/391	25.83	21.47–30.19	81/2,334	3.47	2.73–4.21
	≥51	16/158	10.13	5.37–14.88	45/186	24.19	17.98–30.41	23/419	5.49	3.30–7.68
	χ2	83.091			7.447			277.402		
	*P* value	<0.001			0.114			<0.001		
Females	≤20	9/97	9.28	3.40–15.16	36/60	60.00	47.24–72.76	71/234	30.34	24.41–36.28
	21–30	37/886	4.18	2.86–5.50	300/692	43.35	39.65–47.05	424/6,848	6.19	5.62–6.76
	31–40	8/733	1.09	0.34–1.85	282/706	39.94	36.32–43.56	249/9,571	2.60	2.28–2.92
	41–50	4/308	1.30	0.03–2.57	119/286	41.61	35.86–47.36	76/2,393	3.18	2.47–3.88
	≥51	3/111	2.70	0.00–5.77	46/135	34.07	25.98–42.17	15/450	3.33	1.67–5.00
	χ2	28.141			13.171			522.609		
	*P* value	<0.001			0.010			<0.001		

Analysis of pathogen co-infection trends across age groups revealed that males exhibit a concentration of risk in youth with a significant decline with age, whereas trends in females are slightly different. In males, prevalence of all pathogen combinations peaked in the ≤20 age group and differed significantly across age groups—most notably for NG+UU (18.81% [19/101]). Trends in females varied by pathogen combination. UU+CT co-infection prevalence declined significantly with age, with an exceptionally high rate in ≤20 years (36.00% [18/50]). NG+UU and NG+CT also had their highest prevalence in the ≤20-year group, but both showed no statistically significant differences across age groups. Triple infection (NG+UU+CT) in females was very rare across all ages with no significant trend (Table 6).

**TABLE 6:** Age-specific prevalence of co-infection, stratified by sex^*a*^

STD type	Age (years)	Males			Females		
		Positive cases/*n*	Prevalence (%)	95% CI	Positive cases/*n*	Prevalence (%)	95% CI
NG+UU	≤20	19/101	18.81	11.06–26.57	2/27	7.41	0.00–17.96
	21–30	25/737	3.39	2.08–4.70	7/321	2.18	0.57–3.79
	31–40	9/480	1.88	0.66–3.09	2/284	0.70	0.00–1.68
	41–50	6/151	3.97	0.82–7.12	1/122	0.82	0.00–2.44
	≥51	1/85	1.18	0.00–3.52	0/45	0.00	0.00–0.00
	χ2	41.424			7.51		
	*P* value	<0.001			0.111		
NG+CT	≤20	17/142	11.97	6.57–17.38	3/84	3.57	0.00–7.62
	21–30	46/1,213	3.79	2.72–4.87	10/827	1.21	0.46–1.96
	31–40	19/882	2.15	1.19–3.11	3/714	0.42	0.00–0.90
	41–50	9/330	2.73	0.96–4.49	3/297	1.01	0.00–2.15
	≥51	2/155	1.29	0.00–3.09	1/101	0.99	0.00–2.95
	χ2	28.112			6.816		
	*p*	<0.001			0.146		
UU+CT	≤20	18/129	13.95	7.89–20.01	18/50	36.00	22.22–49.78
	21–30	82/1,354	6.06	4.78–7.33	44/592	7.43	5.31–9.55
	31–40	39/1,077	3.62	2.50–4.74	17/636	2.67	1.42–3.93
	41–50	4/353	1.13	0.02–2.24	7/251	2.79	0.74–4.84
	≥51	4/163	2.45	0.05–4.85	3/112	2.68	0.00–5.72
	χ2	44.135			64.88		
	*P* value	<0.001			<0.001		
NG+UU+CT	≤20	8/101	7.92	2.56–13.28	0/26	0.00	0.00–0.00
	21–30	5/734	0.68	0.08–1.28	2/312	0.64	0.00–1.53
	31–40	0/478	0.00	0.00–0.00	1/280	0.36	0.00–1.06
	41–50	1/150	0.67	0.00–1.98	1/120	0.83	0.00–2.48
	≥51	0/85	0.00	–	0/43	0.00	–
	χ2	31.841			1.155		
	*P* value	<0.001			0.886		

^*a*^ “–” indicates that the 95% CI was not estimable due to the presence of groups containing zero observed cases.

*O*/*E* and OR analyses revealed that this age trend did not equate to epidemiological or statistical synergy. In males, the prevalence of NG+UU, NG+CT, and UU+CT all peaked in the ≤20 age group, with *O*/*E* ratios near unity for NG+UU (0.90) and NG+CT (0.92), and above unity for UU+CT (1.50). For NG+UU, the OR was non-significant in ≤20 age group, with suggested possible antagonistic interaction in the 21–30 (OR 0.33, 95% CI 0.21–0.52) and 31–40 age groups (OR 0.26, 95% CI 0.12–0.53; *O*/*E* ratios of 0.50 and 0.38, respectively), and was non-significant in the ≥41 age group. NG+CT followed a similar pattern, with suggested possible antagonistic interaction in the 21–30 (OR 0.59, 95% CI 0.41–0.84) and 31–40 age groups (OR 0.57, 95% CI 0.34–0.96), with *O*/*E* ratios of 0.72 and 0.67, respectively, while the OR was non-significant in the ≤20 and ≥41 age groups. UU+CT differed: suggested possible synergistic interaction was observed in the ≤20 (OR 2.72, 95% CI 1.22–6.04) and 21–30 age groups (OR 1.59, 95% CI 1.18–2.14), with *O*/*E* ratios of 1.50 and 1.31, respectively; the OR was non-significant in the 31–40 and ≥51 age groups; and suggested possible antagonistic interaction was observed in the 41–50 age group (OR 0.33, 95% CI 0.11–0.96, *O*/*E* 0.43). In females, for NG+UU, the OR was non-significant in the 21–30 age group and not estimable in the other age groups due to a zero cell in the 2 × 2 table. For NG+CT, the OR was non-significant in the ≤20 and ≥51 age groups, with suggested possible synergistic interaction in the 21–30 (OR 4.49, 95% CI 2.01–10.03) and 31–40 age groups (OR 16.35, 95% CI 3.51–76.26), with *O*/*E* ratios of 3.01 and 9.00, respectively. The OR was not estimable in the 41–50 age group. For UU+CT, a suggested possible synergistic interaction was observed in the ≤20 (OR 3.90, 95% CI 1.05–14.51) and 21–30 age groups (OR 2.49, 95% CI 1.47–4.22), with *O*/*E* ratios of 1.24 and 1.44, respectively; the OR was non-significant in the 31–40 and 41–50 age groups and not estimable in the ≥51 age group (Table 7).

**TABLE 7:** Age- and sex-specific analysis of co-infection patterns: *O*/*E* ratios and ORs for pathogen combinations^*a*^^,^^*b*^

Sex	Category of detection	Age (years)	Total *n*	Observed co-infection (*n*)	Expected co-infection (*n*)	*O*/*E* ratio	OR (95% CI)	χ²	*P* value
Males	NG+UU	≤20	101	19	21.07	0.90	0.70 (0.31–1.58)	0.731	0.392
		21–30	737	25	49.99	0.50	0.33 (0.21–0.52)	24.109	<0.001
		31–40	480	9	23.58	0.38	0.26 (0.12–0.53)	15.252	<0.001
		41–50	151	6	6.34	0.95	0.92 (0.34–2.48)	0.029	0.866
		≥51	85	1	1.91	0.52	0.43 (0.05–3.72)	0.697	0.404
	NG+CT	≤20	142	17	18.51	0.92	0.80 (0.37–1.70)	0.338	0.561
		21–30	1,213	46	64.28	0.72	0.59 (0.41–0.84)	8.902	0.003
		31–40	882	19	28.30	0.67	0.57 (0.34–0.96)	4.589	0.032
		41–50	330	9	7.64	1.18	1.27 (0.57–2.80)	0.340	0.560
		≥51	155	2	1.55	1.29	1.38 (0.28–6.78)	0.152	0.697
	UU+CT	≤20	129	18	12.00	1.50	2.72 (1.22–6.04)	6.242	0.012
		21–30	1,354	82	62.81	1.31	1.59 (1.18–2.14)	9.537	0.002
		31–40	1,077	39	35.36	1.10	1.16 (0.78–1.72)	0.567	0.451
		41–50	353	4	9.37	0.43	0.33 (0.11–0.96)	4.571	0.033
		≥51	163	4	2.63	1.52	1.91 (0.53–6.91)	0.918	0.338
Females	NG+UU	≤20	27	2	1.63	1.23	–	–	–
		21–30	321	7	4.74	1.48	2.67 (0.68–10.52)	2.164	0.141
		31–40	284	2	0.87	2.29	–	–	–
		41–50	122	1	0.45	2.22	–	–	–
		≥51	45	0	0.00	0.00	–	–	–
	NG+CT	≤20	84	3	2.14	1.40	1.89 (0.36–10.00)	0.552	0.457
		21–30	827	10	3.32	3.01	4.49 (2.01–10.03)	11.245	<0.001
		31–40	714	3	0.33	9.00	16.35 (3.51–76.26)	9.324	0.002
		41–50	297	3	0.12	24.75	–	–	–
		≥51	101	1	0.12	8.42	15.83 (1.11–226.68)	3.026	0.082
	UU+CT	≤20	50	18	14.52	1.24	3.90 (1.05–14.51)	4.381	0.036
		21–30	592	44	30.55	1.44	2.49 (1.47–4.22)	12.14	<0.001
		31–40	636	17	12.22	1.39	1.97 (0.94–4.12)	3.315	0.069
		41–50	251	7	5.54	1.26	1.61 (0.53–4.94)	0.705	0.401
		≥51	112	3	1.02	2.95	–	–	–

^*a*^ “–” indicates that the presence of a zero cell in the 2 × 2 contingency table prevented calculation of the OR.

^*b*^ OR for triple infection (NG+UU+CT) are not shown due to unstable estimates caused by extremely low event rates.

### Distribution of three pathogens across different clinical diagnoses

In males, the prevalence of all three pathogens differed significantly across diagnostic groups. NG prevalence was highly concentrated in the “Urogenital inflammation” group (36.11% [680/1,883]). UU was widespread, with high prevalence in multiple groups, including the “Screening and asymptomatic examination” group (33.63% [188/559]). CT infection was also highest in the “Urogenital inflammation” group (19.00% [612/3,221]). In females, the distribution patterns differed from males. NG prevalence differed significantly across female clinical diagnosis groups. However, pairwise comparisons with Bonferroni correction revealed no significant differences between any two groups, which may be partly attributable to the limited sample sizes in certain subgroups. UU maintained high, stable rates across all groups (32.50% [13/40] to 46.07% [82/178]) with no significant variation. In contrast, CT infection rates showed highly significant differences among groups, with the “Urogenital inflammation” group (7.37% [365/4,951]) being relatively higher (Table 8).

**TABLE 8:** The prevalence of NG, UU, and CT across different clinical diagnoses^*a*^^,^^*b*^

Sex	Clinical diagnoses	NG			UU			CT		
		Positive cases/*n*	Prevalence (%)	95% CI	Positive cases/*n*	Prevalence (%)	95% CI	Positive cases/*n*	Prevalence (%)	95% CI
Males	Urogenital inflammation	680/1,883	36.11^b^	33.94–38.28	583/2,582	22.58^b^	20.97–24.19	612/3,221	19.00^e^	17.64–20.36
	History of adverse pregnancy and childbirth	0/3	0.00^abcde^	–	0/1	0.00^ab^	–	15/1,062	1.41^ab^	0.70–2.12
	Infertility	0/75	0.00^de^	–	6/22	27.27^ab^	7.06–47.48	129/9,473	1.36^b^	1.13–1.60
	Non-inflammatory genitourinary diseases	4/39	10.26^ce^	0.29–20.22	24/81	29.63^ab^	19.47–39.79	7/146	4.79^acd^	1.29–8.30
	Screening and asymptomatic examination	9/568	1.58^ad^	0.55–2.61	188/559	33.63^a^	29.70–37.56	129/3,136	4.11^d^	3.42–4.81
	Others	12/244	4.92^acde^	2.19–7.65	48/156	30.77^ab^	23.45–38.09	38/405	9.38^c^	6.53–12.23
	χ2	489.806			33.217			1,542.849		
	*P* value	<0.001			<0.001			<0.001		
Females	Urogenital inflammation	44/1,271	3.46^a^	2.46–4.47	471/1,123	41.94^a^	39.05–44.83	365/4,951	7.37^d^	6.64–8.10
	History of adverse pregnancy and childbirth	0/24	0.00^a^	–	18/47	38.30^a^	23.87–52.72	14/491	2.85^ab^	1.37–4.33
	Infertility	0/42	0.00^a^	–	13/40	32.50^a^	17.33–47.67	164/9,277	1.77^b^	1.50–2.04
	Non-inflammatory genitourinary diseases	2/250	0.80^a^	0–1.91	112/270	41.48^a^	35.57–47.40	68/1,371	4.96^ac^	3.81–6.11
	Screening and asymptomatic examination	9/249	3.61^a^	1.28–5.95	82/178	46.07^a^	38.67–53.46	85/1,450	5.86^acd^	4.65–7.07
	Others	6/299	2.01^a^	0.41–3.61	87/221	39.37^a^	32.87–45.86	139/1,956	7.11^cd^	5.97–8.25
	χ2	12.042			3.540			309.256		
	*P* value	0.034			0.617			<0.001		

^*a*^ Prevalence (%) with different superscript letters (a, b, c) has a significant difference (χ2 or Fisher’s, *P* < 0.05).

^*b*^ “–” indicates that the 95% CI was not estimable due to the presence of groups containing zero observed cases.

## DISCUSSION

Based on large-scale patient screening, this study revealed three core epidemiological characteristics of STIs in Guangzhou. Firstly, UU, CT, and NG infections all showed a significant trend toward affecting younger individuals, with the highest prevalence concentrated in individuals aged ≤20 years. Secondly, these three pathogens exhibited distinct gender distribution patterns: UU was predominant in females, while NG and CT showed higher detection rates in males. Thirdly, co-infections demonstrated different epidemiological features. These findings provide a scientific basis for formulating targeted prevention and control strategies.

### Widespread age aggregation

STIs pose a major public health challenge in China, underscored by recent analyses confirming a substantial nationwide burden of infection. Evidence indicates that NG, UU, and CT infections are highly prevalent among outpatients, with the highest burden concentrated in adolescents and young adults aged ≤20 years (17). Similarly, extensive screening data reveal that a considerable proportion of the childbearing-age population is infected with at least one pathogen, with the 18–24 age group showing the highest infection rate (18). Critically, beyond causing acute urethritis and cervicitis, these infections are closely linked to severe reproductive health complications, including pelvic inflammatory disease, infertility, and adverse pregnancy outcomes (19). In this study, young individuals (≤20 years) constituted the primary population for NG, UU, and CT infections. This finding is consistent with results from multiple domestic studies in Shanghai, Hangzhou, and Beijing (20–22). It aligns with the global epidemiological observation that the burden of STIs is highest among sexually active adolescents and young adults. The in-depth analysis suggests that the underlying mechanisms involve multiple factors from both behavioral and social perspectives: adolescents and young adults in this age group, being at their peak of sexual activity yet often lacking sufficient awareness of protective measures, face increased exposure opportunities due to this combination of high-risk behavior and limited knowledge (23). This serious situation presents clear demands for public health policy: it is imperative to prioritize sexual health education by promoting standardized programs in schools and communities. Simultaneously, establishing accessible screening service systems to lower barriers for adolescents is crucial for controlling the spread of STIs (23, 24).

### Fundamental differences in gender distribution

The distributions of NG, UU, and CT infections exhibited distinct patterns by sex. In this study, a significantly higher prevalence of NG was observed in males (25.07%) than in females (2.86%). CT prevalence showed a comparable male predominance (5.33% in males vs 4.28% in females), which is well-documented in other Chinese cohorts. Data from Shanghai indicate that the prevalence of NG and CT in males is approximately 7.2 and 1.8 times that of females, respectively (20). Similarly, research from Hangzhou reports that the prevalence of NG and CT in males was about 6.3- and 1.6-fold higher than in females (25).

This persistent regional pattern stands in contrast to the results of population-based national surveys in the United States (U.S.), which indicate a higher burden of both NG and CT infections among women compared to men (26, 27). This discrepancy stems from the combined effect of healthcare practices, biological characteristics, and the accessibility of laboratory technology. Primarily, the U.S. Centers for Disease Control and Prevention guidelines advocate for routine screening in sexually active young women, enabling the systematic detection of more occult cases (1). In contrast, China currently lacks large-scale asymptomatic screening programs, making the detection of clinical cases more reliant on patient-initiated healthcare visits. Concurrently, NG and CT infections in males often present with acute and typical symptoms, which significantly increase their healthcare-seeking rates. Conversely, female infections are frequently asymptomatic or exhibit atypical symptoms; in the absence of active screening, the reported clinical infection rates in males are consequently significantly higher than in females (2). Additionally, variations in laboratory testing technology may further reduce the probability of detecting infections in women, particularly those who are asymptomatic. These factors collectively contribute to the discrepancy between our data and international reports.

In this study, NG and CT infections in males were highly concentrated in the “urogenital inflammation” diagnostic group (36.11% and 19.00%, respectively), demonstrating a typical symptom-driven pattern. This aligns with the role of males as “sentinel” cases for STIs: the anatomical features of the male urethra lead to prominent symptoms such as dysuria and urethral discharge upon infection, prompting timely medical consultation (2). In contrast, NG and CT infections in females presented a dual pattern of both symptomatic and occult infections (28). Our study found that CT infection in females maintained notably high prevalence in the urogenital inflammation (7.37%), others (7.11%), and screening and asymptomatic examination (5.86%) groups, while NG infection was clearly detected in both the inflammation (3.46%) and screening (3.61%) groups. Consequently, case detection in women relies more heavily on active screening. This epidemiological reality calls for a dual focus in public health response: enhancing etiological diagnosis in symptomatic men remains crucial, while for women, the cornerstone of prevention must be the expansion of active screening programs to effectively avert severe sequelae such as pelvic inflammatory disease and infertility (29).

In contrast to the marked male predominance observed for NG and CT infections, UU, as the most frequently detected pathogen overall in this study (30.91%), exhibited a stable pattern of higher prevalence in females (41.67% vs 24.96% in males), with the infection rate being particularly prominent among females aged ≤20 years. This female-biased biological distribution pattern is supported by other Chinese reports (20, 22). In this study, UU prevalence in males was high in both the “Screening and asymptomatic examination” and “Others” groups. Conversely, females maintained high and stable prevalence across all diagnostic groups, with infection rates consistently higher than those in males across all age strata. These observations corroborate the established biological nature and international consensus that UU commonly exists as a commensal organism in the urogenital tract, with especially high carriage rates observed in asymptomatic healthy women. UU can colonize the female reproductive tract, causing asymptomatic infections. In men, UU can also colonize the urethra, but infection, such as urethritis, typically occurs only when the bacterial load exceeds a certain threshold (30–32). The European STI Guidelines Editorial Board explicitly notes that asymptomatic carriage of UU is common and, unless a high bacterial load is present, it is unlikely to be a causative agent; therefore, routine testing for UU in asymptomatic individuals is not recommended (33). This stance corresponds with the 2025 Chinese Expert Consensus, which states that asymptomatic carriage of UU is very prevalent, with most carriers not progressing to disease; the pathogenicity of UU primarily manifests at high bacterial loads. Consequently, the consensus does not endorse routine UU screening for asymptomatic populations, suggesting testing only for symptomatic individuals after excluding other pathogens. Treatment decisions should be strictly grounded in clinical symptoms and, whenever possible, informed by quantitative bacterial load measurements (2, 34).

Nevertheless, the pathogenic potential of UU warrants attention in specific clinical contexts. Research indicates that UU infection is associated with non-gonococcal urethritis and a decline in semen quality parameters in men, representing a potentially modifiable factor in male infertility (35). In women, UU triggers a signaling cascade through a specific adhesion mechanism, ultimately leading to inflammatory responses and tissue damage, and has been linked to bacterial vaginosis and pelvic inflammatory disease (36). During the perinatal period, vaginal colonization with UU is a well-established risk factor for premature rupture of membranes, preterm birth, chorioamnionitis, and neonatal pulmonary diseases (37). Therefore, the UU data from this study convey a dual public health implication. For UU detected in individuals undergoing health check-ups or presenting with non-specific symptoms, clinicians should adhere to guidelines to avoid overtreatment and reduce unnecessary antibiotic use (38). For patients with clear symptoms of urogenital tract inflammation, infertile couples, or those with a history of adverse pregnancy outcomes, UU should be incorporated into the differential diagnosis. Combining culture methods and susceptibility testing can aid in distinguishing colonization from true infection, thereby enabling precise management of this pathogen (39).

In this study, the prevalence of NG showed an overall declining trend in both males and females from 2020 to 2024, which is consistent with reports from Hangzhou (25). This trend parallels the implementation of strengthened STI control measures and antibiotic stewardship, alongside the impact of the COVID-19 pandemic (40). For CT, the observed reduction during the pandemic phase is consistent with findings from the Western China outpatient cohort, in which CT positivity also declined markedly from the pre-pandemic to the pandemic period (41). The significant decline in NG and CT positivity observed during 2022–2023 coincided with the period of stringent COVID-19 containment measures in China. The social restrictions during this period likely reduced sexual partner change rates, thereby interrupting transmission chains; concurrently, reduced testing led to a decrease in the number of reported cases (42). Furthermore, the rebound in testing volume and the potential resurgence of infections following the lifting of restrictions in early 2023 highlight the importance of sustained public health measures. In males, however, UU detection declined in 2023 alongside NG and CT, whereas in females, UU detection remained high despite the pandemic, consistent with its role as a common commensal whose established colonization may be less susceptible to transient behavioral changes. This sex-specific pattern merits further investigation. Therefore, greater emphasis should be placed on symptom-based, precise diagnosis and treatment for the prevention and control of UU (33).

### Patterns of co-infection

This study found that UU+CT co-infection was the most common. This aligns with conclusions from multiple studies in Hangzhou, Beijing, and Southwest China (22, 25, 43). Moreover, the rate of UU+CT co-infection showed no statistical difference between genders (4.78% in males vs 5.42% in females). As a conditional pathogen, UU infection may be more inclined to cause non-destructive mucosal colonization and inflammation, altering the microenvironment to facilitate CT invasion. Some research also suggests that pre-existing CT infection might alter the local cervical immune environment, thereby promoting persistent colonization by other microorganisms, such as UU (44). Critically, our *O*/*E* and OR analyses suggested a synergistic epidemiological interaction for this pathogen pair in both young males and females (≤30 years), consistent with the facilitative linkage described above. The antagonistic interaction of UU+CT in men aged 41–50 years may reflect instability at low prevalence rather than biological exclusion. Therefore, concurrent screening for CT is necessary for males at high risk for UU infection, such as those with symptoms of non-gonococcal urethritis or infertility. For females at high risk for CT infection, UU should also be considered for inclusion in routine screening, especially when clinical manifestations like genital tract inflammation or infertility are present.

For NG+UU, prevalence in males was significantly higher than in females (3.86% in males vs 1.50% in females). Prevalence peaked in ≤20-year-olds of both sexes, but the OR was non-significant in this stratum, indicating that the high crude co-infection rate reflected high singleton prevalences rather than synergy. In males, *O*/*E* and OR analyses revealed an antagonistic interaction in 21–30-year-olds and 31–40-year-olds, while the OR was non-significant in ≥41-year-olds. This antagonistic pattern in males aged 21–40 years aligns with findings from Hangzhou (25). In the male urethra, NG infection is characterized by acute neutrophilic inflammation with epithelial exfoliation and barrier disruption (45), and UU is known to reside in the male lower urogenital tract and to adhere to urethral epithelial cells (32). The physical loss of epithelial cells from the urethral surface during gonococcal urethritis may therefore reduce the available attachment niche for UU and may plausibly contribute to the antagonistic NG+UU interaction seen in men aged 21–40 years in the present data. Future multi-center clinical studies are needed to provide a more comprehensive epidemiological picture of NG+UU co-infection.

NG+CT co-infection in this study showed a significant gender difference, with a higher rate in males than in females (3.42% vs 0.99%), consistent with prior reports (20, 46). *O*/*E* and OR analyses revealed that, in both males and females, the OR was non-significant among ≤20-year-olds, also suggesting that the peak crude NG+CT prevalence in this age group reflected high singleton prevalences of NG and CT rather than synergy. The antagonistic NG+CT interaction observed in men aged 21–40 years may share a similar mechanism to that proposed for NG+UU, whereby NG-induced inflammation and epithelial exfoliation reduce available attachment sites for concurrent pathogens. In females aged 21–40 years, by contrast, a synergistic interaction was observed despite low co-infection prevalence, suggesting a sex-specific divergence from this mechanism. Further experimental studies are needed to elucidate the biological underpinnings of both the antagonistic and synergistic interactions observed in different sexes.

Furthermore, our data revealed a significant age-related difference among males in the prevalence of NG+UU+CT triple infection, with the highest detection rate observed in those aged ≤20 years (7.92%). In contrast, the prevalence in females remained consistently low across all age groups (0%–0.83%), with no significant age variation. This distribution pattern corresponds with the established epidemiology of high-risk sexual behaviors, where young males’ sexual practices may lead to increased exposure to multiple pathogens (47). These findings highlight the importance of considering triple infections when diagnosing and managing sexually transmitted diseases in young male populations, while also suggesting the need for sex-specific prevention strategies.

In summary, UU+CT showed high co-infection prevalence and a synergistic interaction in both sexes aged ≤30 years, suggesting a facilitative epidemiological relationship in young adults. In contrast, for NG+UU and NG+CT, the peak crude prevalence in ≤20-year-olds of both sexes likely reflected high singleton prevalences rather than synergy, as no significant interaction was detected in this age group. The divergent interaction patterns across pathogen pairs and age groups underscore the complexity of co-infection epidemiology. These statistically derived interactions confirm that the observed co-infection trends are not merely artifacts of independent age-dependent prevalence. However, these *O*/*E* and OR estimates reflect epidemiological associations; whether they correspond to direct biological mechanisms of competition, facilitation, or immune modulation requires further experimental investigation.

Therefore, the epidemiological characteristics revealed in this study provide a key scientific basis for formulating evidence-based and targeted STI prevention and control strategies. However, any strategy aimed at improving treatment success and interrupting transmission must confront and effectively address the complex and evolving challenge of pathogen antimicrobial resistance. Currently, the World Health Organization has classified the increasing resistance of NG to first-line agents as a serious public health problem (48). This challenge is immediate in China, where recent national genomic surveillance has confirmed the emergence and circulation of high-risk clones harboring the *penA-60.001* gene, conferring high-level ceftriaxone resistance, particularly in southern regions (49, 50). Therefore, the establishment of enhanced regional surveillance and alert systems focused on such resistant lineages is important for informing the development of context-appropriate treatment guidelines and for supporting efforts to mitigate the spread of resistant NG. In contrast, genomic evidence for resistance to first-line antimicrobials, macrolides, tetracyclines, and quinolones in CT has not been established (51, 52). UU presents a distinct profile. Multicenter surveillance data from China indicate that UU has developed notable resistance to fluoroquinolone antimicrobials such as levofloxacin, yet maintains relatively high susceptibility to tetracycline-class drugs, including doxycycline (53). Surveillance among college-aged women in the U.S. shows a low overall rate of resistance; however, the first levofloxacin-resistant strain harboring specific mutations has been detected (54). Therefore, the high prevalence observed in our study underscores the need for enhanced regional surveillance and a strict “symptom-oriented” approach to antibiotic stewardship to curb the selection pressure for resistant lineages. The failure to provide antimicrobial susceptibility data for the isolates was a limitation of this study.

### Study limitations

Several limitations should be acknowledged. First, this was a single-center investigation, which may have introduced selection bias and may not fully represent the general population, thereby potentially limiting the generalizability of the findings. Additionally, the retrospective nature of the data precludes causal inferences. Second, the reliance on clinician-driven testing introduces inherent selection bias; while we adhered to national guidelines, we were unable to access detailed clinical decision-making records. This prevented us from precisely quantifying the adherence of each test to specific guideline provisions or fully adjusting for unmeasured confounders. The inherent challenges in addressing these potential confounders may have resulted in inaccurate risk estimations. Third, the sample sizes in certain subgroups were relatively small, which may have led to unstable estimates of infection rates. Finally, data on strain-specific antimicrobial susceptibility, sexual orientation, and other behavioral variables were lacking. Future multicenter studies incorporating larger sample sizes, comprehensive behavioral data collection, and robust antimicrobial susceptibility surveillance are warranted to address these limitations.

### Conclusion

In summary, this study, based on a large cohort of 37,680 patients in Guangzhou, provided a refined epidemiological profile of NG, UU, and CT infections. First, NG and CT predominated in males (25.07% and 5.33%, respectively), whereas UU was female-predominant (41.67% vs 24.96% in males). Age-specific peaks were observed, with the highest NG prevalence in young males (≤20 years, 48.34%) and the highest UU prevalence in young females (≤20 years, 60.00%). UU+CT emerged as the predominant co-infection pattern, particularly in adolescent females, with a statistically synergistic interaction observed in both sexes aged ≤30 years. Clinical stratification revealed that in males, NG and CT infections exhibited a classic “symptom-driven” pattern. In females, the epidemiological picture is more complex: CT infection maintained notably high prevalence in the urogenital inflammation (7.37%), others (7.11%), and screening and asymptomatic examination (5.86%) groups, while NG was detected in both the inflammation (3.46%) and screening (3.61%) groups, together revealing a significant dual burden of symptomatic and occult infections. UU showed exceptionally high and stable prevalence across all female clinical groups (32.50%–46.07%) and similarly high prevalence across most male groups (22.58%–33.63%), suggesting its predominantly commensal role. These findings emphasized the need for tailored screening strategies: strengthen NG- and CT-focused screening for young symptomatic males, especially those with urogenital inflammation. For females, it is imperative to expand proactive screening for CT and NG to uncover occult infections. Concurrently, it is crucial to systematically explore and optimize screening strategies to address the current gap in detecting asymptomatic infections among women. For UU, adherence to clinical guidelines for the implementation of “symptom-oriented” precision management and attention to UU+CT co-infection assessment in adolescent females is advised. Furthermore, our results supported the development of gender-specific diagnostic protocols that distinguish between symptomatic management in males and active case-finding in females to effectively address both overt and hidden infection burdens. Our study delineated the epidemiological profile of STIs in the Guangzhou area and provided evidence for tailored control strategies.

## Data Availability

Due to patient privacy and ethical restrictions of the medical records analyzed in this retrospective study, the de-identified data set extracted from the hospital Laboratory Information System (LIS) cannot be deposited in a public repository. The data that support the findings of this study are available from the author upon reasonable request, subject to approval by the Ethics Committee of the Affiliated Guangdong Second Provincial General Hospital of Jinan University. No new sequencing data were generated.
